# Cardio-Hepatic Interaction in Cardiac Amyloidosis

**DOI:** 10.3390/jcm13051440

**Published:** 2024-03-01

**Authors:** Sandra Michaela Ihne-Schubert, Oliver Goetze, Felix Gerstendörfer, Floran Sahiti, Ina Schade, Aikaterini Papagianni, Caroline Morbach, Stefan Frantz, Hermann Einsele, Stefan Knop, Claudia Sommer, Beat Müllhaupt, Torben Schubert, Stefan Störk, Andreas Geier

**Affiliations:** 1 Interdisciplinary Amyloidosis Center of Northern Bavaria, University Hospital Würzburg, 97080 Würzburg, Germany; 2 Department of Internal Medicine II, Hematology, University Hospital Würzburg, 97080 Würzburg, Germany; 3 CIRCLE—Centre for Innovation Research, Lund University, 22100 Lund, Sweden; 4 Department of Internal Medicine IV, University Hospital Gießen and Marburg, 35392 Gießen, Germany; 5 Department of Internal Medicine II, Hepatology, University Hospital Würzburg, 97080 Würzburg, Germany; 6 Department of Medicine, Universitätsklinikum Knappschaftskrankenhaus Bochum, 44892 Bochum, Germany; 7 Comprehensive Heart Failure Center, University Hospital Würzburg, 97080 Würzburg, Germany; 8 Department of Internal Medicine I, Cardiology, University Hospital Würzburg, 97080 Würzburg, Germany; 9 Department of Thoracic Surgery and Thoracic Endoscopy, Helios Klinikum Erfurt, 99089 Erfurt, Germany; 10 Department of Neurology, University Hospital Würzburg, 97080 Würzburg, Germany; 11 Department of Internal Medicine V, Hospital Nürnberg Nord, 90419 Nürnberg, Germany; 12 Department of Gastroenterology and Hepatology, University Hospital Zurich, 8091 Zurich, Switzerland; 13 Fraunhofer Institute for Systems and Innovation Research ISI, 76139 Karlsruhe, Germany

**Keywords:** cardiac amyloidosis, ^13^C-methacetin breath test (MBT), liver stiffness, vibration-controlled transient elastography (VTCE), PDR_peak_, congestion

## Abstract

**Background:** Congestion is associated with poor prognosis in cardiac amyloidosis (CA). The cardio-hepatic interaction and the prognostic impact of secondary liver affection by cardiac congestion in CA are poorly understood and require further characterisation. **Methods:** Participants of the amyloidosis cohort study AmyKoS at the Interdisciplinary Amyloidosis Centre of Northern Bavaria with proven transthyretin (ATTR-CA) and light chain CA (AL-CA) underwent serial work-up including laboratory tests, echocardiography, and in-depth hepatic assessment by vibration-controlled transient elastography (VCTE) and ^13^C-methacetin breath test. **Results:** In total, 74 patients with AL-CA (n = 17), ATTR-CA (n = 26) and the controls (n = 31) were analysed. ATTR-CA patients showed decreased microsomal liver function expressed by maximal percentage of dose rate (PDR_peak_) related to hepatic congestion. Reduced PDR_peak_ in AL-CA could result from altered pharmacokinetics due to changed hepatic blood flow. Liver stiffness as a combined surrogate of chronic liver damage and congestion was identified as a predictor of all-cause mortality. Statistical modelling of the cardio-hepatic interaction revealed septum thickness, NT-proBNP and PDR_peak_ as predictors of liver stiffness in both CA subtypes; dilatation of liver veins and the fibrosis score FIB-4 were only significant for ATTR-CA. **Conclusions:** Non-invasive methods allow us to characterise CA-associated hepatic pathophysiology. Liver stiffness might be promising for risk stratification in CA.

## 1. Introduction

Systemic amyloidosis represents a complex multi-system disorder that is caused by the deposition of misfolded proteins in the tissue leading to organ dysfunction. The prognosis depends largely on the presence and severity of cardiac involvement [1,2,3,4,5,6]. Transthyretin (ATTR) and systemic light chain (AL) amyloidosis are the most common forms in general, as well as in the subtype of cardiac amyloidosis (CA). Mechanical and local cytotoxic effects can be found as underlying pathogenetic mechanisms in both forms [1,2]. In cardiac light chain amyloidosis (AL-CA), circulating free light chains exert additional direct cardiotoxic effects by direct activation of a MAPK pathway as the main reason for cardiotoxicity with direct influence on NT-proBNP [7,8,9].

Irrespective of the subtype of CA, elevated liver function tests are common. They are, however, usually neither consequences of primary liver disorders nor hepatic amyloidosis, as hepatic involvement does not occur in ATTR in general and only in about 15% of AL amyloidosis [10]. Therefore, observed elevation of liver functional tests most likely results from cardiac congestion and is thus to be considered secondary. In the light of this, the current study aims to

(i). Non-invasively characterise and model secondary liver affection (dynamic hepatic function and tissue elasticity) in patients with CA;
(ii). Evaluate the diagnostic and prognostic utility of quantitative dynamic liver function tests and vibration-controlled transient elastography (VCTE) regarding mortality.

## 2. Materials and Methods

### 2.1. Study Population

The Amyloidosis Cohort Study (AmyKoS) recruits consecutive patients presenting to the Interdisciplinary Amyloidosis Center of Northern Bavaria, Würzburg, Germany, with suspected or proven amyloidosis. This study complies with the Declaration of Helsinki and received positive votes from the Medical Ethics Committee at the Julius-Maximilians-University of Würzburg (48/18). All participants provided written informed consent. For the present analysis, participants with proven cardiac and excluded hepatic AL (AL-CA), according to Gertz et al. in 2005, as well as proven cardiac ATTR amyloidosis (ATTR-CA), were identified [11]. Further, control patients from the AmyKoS stock with excluded cardiac and hepatic amyloidosis manifestations entered the analysis, ensuring an identical diagnostic work-up by the same investigators and in the same setting. The subtype-spanning approach was chosen to generate generalisable results that reflect the different pathomechanisms.

All patients underwent a serial detailed work-up, including extensive laboratory tests, standardised transthoracic echocardiography, VCTE to assess liver stiffness (in kPA) and ^13^C-methacetin breath test (MBT) for microsomal liver function expressed by PDR_peak_. Laboratory parameters were obtained from routine blood analysis according to locally established standards.

The well-established fibrosis score FIB-4 was calculated according to the generally applicable formula [12,13,14]:

$$FIB-4=\frac{age\;[years]\times AST\;[\frac{U}{L}]}{platelet\;count\;[\frac{10^{9}}{L}]\times \sqrt{ALT[\frac{U}{L}]}}$$

FIB-4 was cleaned for its statistical component related to congestion by regressing the score on NT-proBNP and liver vein congestion and using the residuals from the regression as a congestion-filtered version of the score (indicated by the subscript “clean”; FIB-4_clean_). 

Microsomal function measured by MBT over 1 h of breath sampling after ingestion of 75 mg 4′-O-^13^C-methacetin in a fasted state (Euriso-top, 91194 Saint-Aubin Cedex, Côte-d’Or, France; chemical purity of 99.7% and an isotopic purity of 99.1%) dissolved in 100 mL water was expressed by the maximum percentage dose rate PDR_peak_ (%/h), since the maximum ^13^CO_2_ excretion is least affected by post-CYP1A2 processes, such as loss through exchange reactions, e.g., with the bicarbonate pool or integration into the skeletal system [15]. This is in line with the European guideline on indications, performance and clinical impact of ^13^C-breath tests in adult and paediatric patients [16]. Explanations regarding the applied methods can be found in the Supplementary Materials (Table S1 [12,13,14,16,17,18,19,20,21,22]). Moreover, detailed test descriptions are given elsewhere [23].

### 2.2. Independent Reference Groups for Microsomal Liver Function

The raw data of young healthy participants and patients with chronic hepatitis C infection (mean age 46.2 years, SD 11.3 years, range 20–74 years) with low histological fibrosis stages [23] were provided by O. G. and B. M. and reanalysed regarding microsomal function within different stages of fibrosis and inflammation using PDR_peak_ as a marker of interest (for the rationale, see above). Additional reference groups were identified via PubMed search using the general keyword “methacetin breath test” [23,24,25,26,27,28,29]. The minimum requirements for the use as a reference group were the clear definition of the reported collective by a single underlying disorder and the specification of mean, standard deviation and the total number of patients analysed for PDR_peak_ to enable comparison with the CA patients. 

### 2.3. Statistical Model Development and Analysis

Since our goal was to characterise secondary liver affection in CA, we first performed a mean value comparison of the two subtypes of amyloidosis and the control in our sample, as well as with different groups of primary liver affection (see reference groups for microsomal liver function) regarding significant differences by *z*-test. Furthermore, the pairwise non-parametric Spearman correlation analysis was performed, referring to the cross-sectional data set of all patients at first evaluation to obtain a first impression of the prognostic value of the different liver function tests, as well as their relation to congestion. 

In the second step, we focused on the effects of congestion expressed by the surrogates NTproBNP and liver vein dilatation on the hepatic metabolic (microsomal) activity expressed by PDR_peak_ and chronic liver damage, specifically fibrosis, expressed by the lab-based score FIB-4. We used multivariate linear regression analysis for the evaluation based on the panel data set resulting from a serial work-up of patients.

As FIB-4 and PDR_peak_ are only valid surrogate parameters for parts of the pathomechanisms behind secondary liver affection, we chose liver stiffness as a clinically easily accessible and well-established summatory surrogate parameter for chronic liver damage going beyond fibrosis.

In the third step, we then specifically modelled the factors influencing liver stiffness. Based on a theoretical model, which we adapted from Müller et al. [30] to the specific situation of CA, we chose the following five parameters in the final model explaining liver stiffness: septum thickness, NT-proBNP, dilatation of liver veins, PDR_peak_ and FIB-4_clean_. To test the effects of these theoretically derived parameters on liver stiffness, a regression approach was employed. While principally ordinary linear regression would be feasible, one problem is that further parameters may be of relevance. We therefore decided to implement a data-driven regression model, which is able to select additional parameters depending on their explanatory power. The formula to describe the statistical model can be written as follows:

$$y_{it}=\sum_{j=1}^{J}x_{itj}\beta_{j}+\sum_{k=1}^{K}c_{itk}\delta_{k}+u_{it}$$

y_it_—log stiffness;
x_it_—core variables (septum thickness, NT-proBNP, dilatation of liver veins, PDR_peak_ and FIB-4_clean_);
c_it_—high-dimensional control variables;
β_j_—coefficients of the variables of interest;
δ_k_—coefficients of the high-dimensional control variables;
u_it_—disturbance term;
J, K—number of variables, K can become very large.

To systematically identify the relevant control variables in our high-dimensional data set and to avoid a bias due to manual pre-regression selection of control variables, a post-cluster least absolute shrinkage and selection operator regression (LASSO), following Belloni et al. (2016) [31], was performed based on the panel data set including all available repetitive data sets of the described patient population.

In the fourth and final step, we analysed the survival among our cohort by calculating Kaplan–Meier curves and assessed the prognostic value of liver stiffness in comparison to the other two hepatic markers FIB-4 and PDR_peak_, as well as established cardiac biomarkers for mortality. We used a Cox regression approach with time-varying data, which expresses the mortality risk by the hazard rate, i.e., the time-normalised risk of death as a function of key explanatory variables $x_{it}$:

$$h\left(t,x_{it}\right)=h_{0}(t)\cdot \exp\left(x_{it}\beta \right)$$

β is a vector of associated regression parameters. $h_{0}(t)$ is the unknown baseline hazard defining the baseline mortality risk if all parameters $x_{it}$ are zero. The baseline hazard is not estimated but treated as noise in the regression. The interest then lies in estimating β, which determines the direction of the influence each clinical parameter has on the hazard function. For example, if for a parameter the associated element of β is larger than zero, the influence on the hazard function is positive. In the regressions, for interpretative convenience, we do not report the raw coefficients, but their exponentiated versions, because the exponentiated coefficients can be interpreted as hazard rates. The neutral point (β=0) is then 1 because $\exp\left(0\right)=1$. Also, if an estimated hazard rate is then, for example, equal to, say, 1.05, it means that a one-unit increase in a parameter increases the mortality risk in a fixed time interval by 5%. Cox regression has a number of desirable features, which make it preferable over Full Maximum Likelihood survival models. Specifically, it is semi-parametric in the sense that it does not impose parametric functional assumptions on the baseline hazard. Results were visualised by forest plots.

All statistical analyses were performed using STATA^®^ version 14.

## 3. Results

From November 2017 until April 2020, 74 patients with AL-CA (n = 17), ATTR-CA (n = 26) and the controls (n = 31) with, in total, 177 observations, were evaluated. The basic characterisation of the cohort is summarised in Table 1. The mean age of ATTR-CA, AL-CA and controls was 74.9 ± 7.2, 64.0 ± 8.1 and 63.1 ± 1.6 years, respectively.

### 3.1. Characterisation of the Cardiac Function within the Cohort

In total, 76.9% of patients with ATTR-CA and 71.4% of those with AL-CA presented in NYHA functional class II or higher compared to 41.9% of the control patients. AL-CA and ATTR-CA patients showed the typical clinical picture of heart failure with preserved ejection fraction (HFpEF), but LVEF among ATTR-CA was significantly lower compared to AL-CA and controls within the established range for HFpEF of LVEF ≥ 50%. Septum and posterior wall thickness were significantly increased in CA, in ATTR-CA more than in AL-CA. Cardiac biomarker levels such as NT-proBNP and high-sensitive troponin T levels were also significantly elevated in both, but in AL-CA more than ATTR-CA. Diastolic function was significantly impaired in CA compared to the controls. Signs of hypervolemia were highly prevalent in CA (Table 1 and Table S2). The median daily dosage of diuretics among those treated with diuretics was equivalent to 17.5 (10.0; 20.0) mg torasemide among ATTR-CA, 30.0 (16.25; 55.00) mg among AL-CA and 12.5 (9.38; 16.25) mg among controls, respectively. More details are summarised in Table 1 and Table S2. 

### 3.2. Characterisation of Hepatic Affection in CA 

Mean values of AP were normal. But, in comparison to control patients, AP was elevated in ATTR-CA (*p* < 0.1). AP elevation met in 7% of cases of ATTR-CA formally the definition of hepatic involvement according to Gertz et al., 2005 [11]. Mean GGT levels were elevated in both ATTR-CA and AL-CA, but reached significance only in ATTR-CA compared to the control group and AL-CA. Transaminases and GLDH were normal in all groups. Static liver synthetic function expressed by cholinesterase was reduced in both AL-CA and ATTR-CA compared to the controls, in AL-CA slightly more than in ATTR-CA. In analogy, PDR_peak_ was reduced in both forms of CA, but reached statistical significance only in AL-CA.

Moreover, AL-CA patients showed predominantly a similar stiffness compared to the control group, whereas in ATTR-CA the mean stiffness was increased to 7.9 ± 4.5 kPa, which is formally in the range of F1/F2 fibrosis.

Using young healthy adults as the reference group, we found significantly lower PDR_peak_ levels in CA. The decrease was more pronounced in AL-CA than in ATTR-CA (Table S3). Analogously, the PDR_peak_ reduction was also significant, albeit less pronounced, in comparison with a healthy elderly collective. A regression regarding age in the presented CA collective showed congruent results. The extent of the PDR_peak_ reduction was comparable to findings in patients with chronic hepatitis C and those with histologically significant inflammation or fibrosis (Supplement Table S3). Moreover, the PDR_peak_ levels of patients with alcohol-induced cirrhosis Child–Pugh A or primary biliary cholangitis with LSS III were similar.

### 3.3. Effect of Cardiac Congestion on Microsomal Liver Function Expressed by PDR_peak_

To get an impression of the effect of cardiac congestion on PDR_peak_, a multivariate regression analysis referring to the panel data set was performed (Table 2): We found a significantly lower level of PDR_peak_ in AL-CA, but not in ATTR-CA. NT-proBNP was significantly negatively correlated with PDR_peak_ overall. In the subtype-specific analysis, the effect of NT-proBNP on PDR_peak_ was comparable to the overall effect for ATTR-CA, but in AL-CA, it was negligible. In contrast, liver vein dilatation was associated with a significant decrease in PDR_peak_ in the entire cohort, which was more pronounced and significant in ATTR-CA. This effect could not be calculated in the AL-CA subgroup due to multicollinearity, which indicates that the effect is comparable to the baseline effect.

To test the hypothesis that the altered pharmacokinetics of ^13^C-methacetin may result from changes in hepatic blood flow due to impaired cardiac function in AL-CA, according to the PK model published by Lane-Parashos et al. in 1986 [32], tr-v_max_ and right atrial volume were chosen as indirect surrogate parameters. A multivariate panel regression analysis showed that tr-v_max_ and right atrial volume were both inversely and significantly correlated with PDR_peak_.

### 3.4. Chronic Liver Damage and Fibrosis in Cardiac Amyloidosis

To assess the role of possible fibrotic processes in CA, we calculated the well-established fibrosis score FIB-4 according to the generally applicable formula (see above in Section 2). At baseline evaluation, 17.6% of AL-CA, 30.7% of ATTR-CA and 0% of controls showed FIB-4 scores > 3.2, a value compatible with a high risk of advanced liver cirrhosis. The increase in FIB-4 was correlated with PDR_peak_, but an apparent threshold for PDR_peak_ with an altered increase in FIB-4 could not be detected. The effect of NT-proBNP and liver vein congestion on FIB-4 was analysed by multivariate panel regression in analogy to the effect on PDR_peak_ because FIB-4 includes AST and ALT as congestion-sensitive parameters (Table 2). Both AL-CA and ATTR-CA showed a significant baseline effect on FIB-4, but there was no significant effect on FIB-4 by NT-proBNP and liver vein dilatation. 

Additionally, the congestion-dependent component of FIB-4 was estimated by linear regression to be between 0.2% in ATTR-CA and 3.7% in AL-CA (overall, 3.0%).

### 3.5. Modelling Liver Affection in Cardiac Amyloidosis

**Figure 1:**
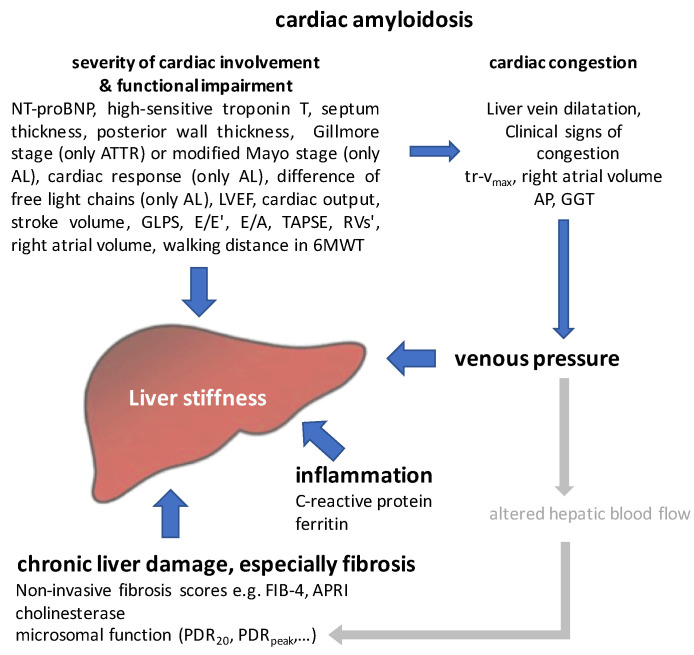
Theoretical model of the cardio-hepatic crosstalk in cardiac amyloidosis (based on the model of Müller et al., 2010 [30] and specifically adapted to cardiac amyloidosis). VCTE is usually applied in patients with a high a priori probability of liver fibrosis due to chronic liver disease and, therefore, stiffness primarily reflects in these patients the grade of liver fibrosis. In contrast, in cardiac amyloidosis, the proposed application of VCTE occurs early in the development of possible fibrosis (so-called cirrhosis cardiaque) and increased stiffness may also result from chronic cardiac congestion. Subtype-spanning main influencing factors for liver stiffness in cardiac amyloidosis are supposed to be the severity of cardiac involvement with resulting impairment of cardiac function, cardiac congestion, inflammation and chronic liver cell damage including fibrosis. Potential clinical, laboratory and instrumental surrogate parameters were assigned based on the literature, their clinical value and availability.

Against the background of the previous results and considerations, we chose liver stiffness as a summatory surrogate for liver affection going beyond fibrosis and adapted the theoretical model proposed by Müller et al., 2010 [30] to the special situation in cardiac amyloidosis (in the absence of hepatic involvement), as shown in Figure 1.

As subtype-spanning main influencing factors the following parameters were defined: severity of cardiac involvement with resulting impairment of cardiac function, cardiac congestion, inflammation and chronic liver cell damage including fibrosis. Clinical, laboratory and instrumental surrogate parameters were assigned to the defined main influencing factors, and the surrogates for the final regression model were selected based on the literature, their clinical value and availability in daily practice, as well as the results of a Spearman correlation analysis (Figure 1; Table S4). High-sensitive troponin and C-reactive protein showed no predictive power for liver stiffness, so these parameters were also subsequently dropped.

**Figure 2:**
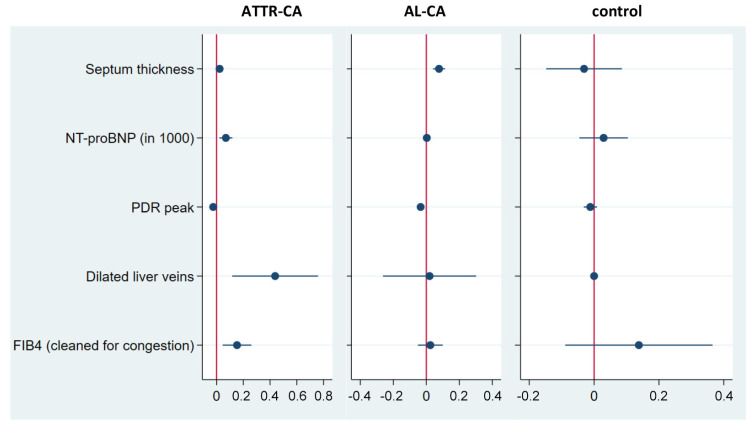
Visualisation of the coefficients of the post-cluster LASSO referring to model 2 (blue dot = regression coefficient; blue lines = 95% confidence intervals).

Based on this, the estimation of the regression model was performed and subgroup-specific coefficients were calculated as shown in Table 3 and visualised in Figure 2. To test the generalisability of the approach and the stability of the results, we estimated the regression model for the subgroup of patients with localised amyloidosis who can be considered as healthy controls (model 1) and for the entire control group, including patients with non-amyloidotic cardiac disorders (model 2).

We were able to prove that septum thickness, NT-proBNP and PDR_peak_ are predictors of liver stiffness in ATTR-CA and AL-CA in both models. Dilated liver veins and FIB-4_clean_ predicted liver stiffness only for ATTR-CA. The inclusion of tr-v_max_ in the models resulted in computational instability (Table S5). However, tr-v_max_ seemed to be a predictor for liver stiffness, but only in AL-CA.

### 3.6. Predictive Value of the Main Influencing Factors Regarding All-Cause Mortality

**Figure 3:**
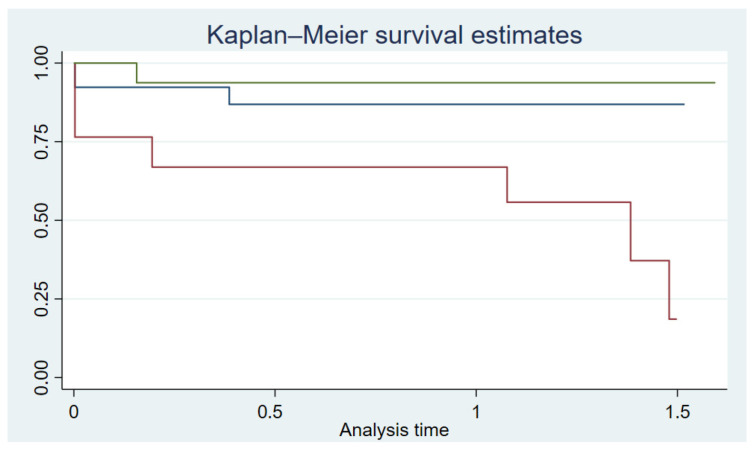
The Kaplan–Meier curves illustrate the overall survival among the three groups (ATTR-CA blue line; AL-CA red line; controls green line). The *X*-axis reflects the observation time in years; the *Y*-axis indicates the proportion of patients still alive.

The median follow-up of the observed patient population was 666 days and, in total, 12 patients died. The Kaplan–Meier survival curves for AL-CA, ATTR-CA and the control group are shown in Figure 3.

The predictive value of liver stiffness and the chosen main influencing factors of our model regarding all-cause mortality were analysed by Cox proportional hazard survival regression. Additionally, in cardiac AL and ATTR amyloidosis, the well-established prognostic marker high-sensitive troponin was added.

**Figure 4:**
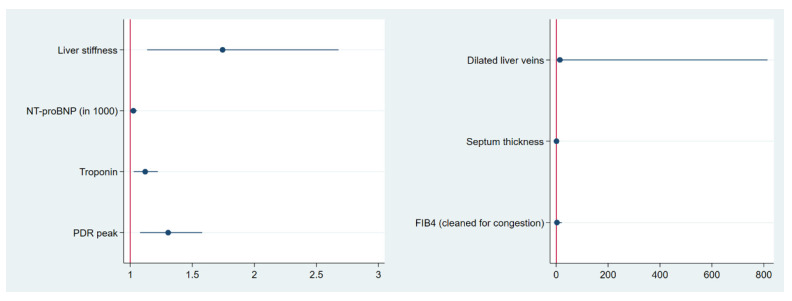
Forest plot for the visualisation of the association between influencing factors and all-cause mortality (blue dot = regression coefficient; blue lines = 95% confidence intervals).

Liver stiffness, high-sensitive troponin, NT-proBNP and PDR_peak_ were significant predictors of all-cause mortality (Table 4; Figure 4). Septum thickness, dilated liver veins and FIB-4_clean_ were not able to predict prognosis.

## 4. Discussion

This study aimed to (1) non-invasively characterise and model secondary liver affection in CA and (2) assess the diagnostic and prognostic value of dynamic liver function tests and VTCE.

In the light of this, the most relevant findings are the following:

- A significant proportion of ATTR-CA patients (7%) in our sample present with AP elevation and therefore formally fulfil the criteria for liver involvement according to Gertz et al., 2005 [11].
- Secondary liver affection in ATTR-CA results in decreased microsomal liver function related to hepatic congestion.
- Reduced PDR_peak_ in AL-CA may result from altered pharmacokinetics due to changed hepatic blood flow.
- Liver stiffness may act as a summatory surrogate for liver affection going beyond fibrosis and also reflects impaired cardiac function in CA, hypervolemia and congestion. Based on this, we were able to model the interaction between liver and heart in ATTR-CA and AL-CA: septum thickness, NT-proBNP and PDR_peak_ have been identified as predictors of liver stiffness for both entities. The dilatation of liver veins and FIB-4_clean_ are significant predictors only in ATTR-CA.
- Liver stiffness, high-sensitive troponin, NT-proBNP and PDR_peak_ are predictors of all-cause mortality, suggesting them as promising factors for risk stratification in cardiac amyloidosis.

So far, little is known about the congestion-related effects of cardiac dysfunction in CA on other organs, especially the liver, particularly the functional (biochemical) and physical (stiffness, matrix deposition) consequences. Moreover, their prognostic relevance is still unclear.

The fact that 7% of the ATTR amyloidosis patients in our sample present with elevated AP levels according to the definition by Gertz et al., 2005 [11] implies that AP is not very specific and may not specifically differentiate between primary and secondary liver affection in AL-CA. AP has to be applied cautiously and examined on a case-by-case basis. More robust and generally applicable parameters for the distinction of primary and secondary liver affection are needed, which can also be supported by the finding that AP seems to identify only part of patients with liver involvement, according to Brunger et al. [33]. Whether or not VCTE and/or compartment-specific ^13^C-breathing tests such as ^13^C-methacetin and ^13^C-methionin breath tests might be helpful in this context has to be investigated in further studies.

Normal levels of transaminases and GLDH do not indicate acute ongoing hepatocyte damage in cardiac amyloidosis. Nevertheless, we were able to show that secondary liver affection is significantly underestimated in the context of CA.

In ATTR-CA, a considerably lower liver synthesis capacity as measured by cholinesterase (*p* < 0.01) and an impaired (albeit not significant) microsomal function as proxied by ^13^C-methacetin breath testing (PDR_peak_) can be observed compared to the control patients. The fact that chronic cardiac impairment negatively impacts hepatic function is not new. Therefore, Malek et al. could demonstrate a significantly impaired metabolic liver function in a small cohort of patients with advanced chronic heart failure [34]. Functional liver mass did not correlate with LVEF, but left atrial diameter did [34]. According to another pilot study published by Hendrichová et al., 2010, the correlation of NT-proBNP with the degree of metabolic liver function impairment did not reach significance in patients with decompensated heart failure [35].

However, the extent of hepatic impairment comparable to an alcohol-induced cirrhosis Child–Pugh A or primary biliary cholangitis with LSS III seems surprising at first glance. However, given that the patients typically develop a renal deterioration in the sense of a cardiorenal syndrome type II in the course of their disease, which is used in the well-established algorithm for risk stratification published by Gillmore et al. [6,36], the observation appears plausible.

In contrast, lower PDR_peak_ levels in AL-CA most likely result in altered blood flow with consecutive changes in pharmacokinetics. This assumption is based on a multivariate panel regression showing that tr-v_max_ and right atrial volume were both inversely and significantly correlated with PDR_peak_. Of course, a direct and invasive measurement of hepatic blood flow by right heart catheter would be desirable to prove this hypothesis, but was not justifiable for patient safety reasons and a known high risk of bleeding in amyloidosis. At the same time, altered blood flow with resulting changes in pharmacokinetics is already known from other confounders such as inflammation, hepatocellular proliferation and hypoxia. Moreover, the high dynamics of cardiac dysfunction in AL-CA have been extensively investigated. The direct cardiotoxicity of circulating free light chains results in an immediate decrease in cardiac function after the infusion of free light chains in animal models such as zebrafish and mouse hearts [7].

In analogy to other chronic liver diseases and given the clinical picture of cirrhose cardiaque, it can be assumed that fibrotic processes may also play a role in this context. Because of the high risk of bleeding in systemic amyloidosis and comparatively low stiffness values that do not justify liver biopsies at this stage of knowledge, a primarily non-invasive approach was chosen for patient safety. As the available non-invasive fibrosis markers are not validated as single direct markers in this context and the established fibrosis scores include at least one congestion-sensitive parameter, e.g., AST, ALT and GGT, we used FIB-4 corrected for the effect of mere congestion (e.g., on the AST to ALT ratio) as a surrogate score for hepatic fibrosis and indicator of increased matrix deposition during CA.

While the clinical assessment of signs of hypervolemia such as oedema, jugular venous congestion, etc., or diuretic use only represents a current snapshot and may be absent in amyloidosis as restrictive cardiomyopathy, liver stiffness might represent a combined surrogate parameter. It might reflect currently detectable hepatic congestion in the context of acute cardiac decompensation, but also chronic effects such as fibrotic processes as “long-term memory” for recurrent damage and repair during acute and chronic cardiac congestion frequently observed in the long-term course of ATTR-CA. These associations were successfully modelled across subtypes in AL-CA and ATTR-CA using state-of-the-art statistical methods. According to the model, hepatic venous congestion and fibrotic processes play a significant role in secondary liver affection in ATTR-CA. Increased liver stiffness may indicate advanced disease stages. Against this background, it is not surprising that liver stiffness serves as an additional predictor of mortality beyond cardiac biomarkers. The negative impact of hypervolemia and recurrent cardiac decompensations is well known for heart failure in general. This also fits with the findings of Gillmore et al. that a reduced eGFR in the sense of cardiorenal syndrome type 2 is prognostically relevant in ATTR-CA and the staging system can be applied serially [6,36].

Considering the direct cardiotoxicity of the free light chains in AL-CA with direct NT-proBNP increase [37], it is conceivable that hepatic venous congestion and fibrosis processes take a background role given the predominance of NT-proBNP in AL-CA.

In light of the presented results and considerations, liver stiffness does not appear to be a useful tool for improving early diagnosis because liver fibrosis due to primary liver disorders is much more frequently found and hepatic congestion is common in heart failure. However, liver stiffness is of prognostic relevance and may indicate advanced stages. While in cardiac AL amyloidosis, high-risk groups with short survival such as Mayo stage IIIB with median survival of < 6 months can be identified based on cardiac biomarkers, the temporal resolution of the staging systems published to date for ATTR-CA is 20–24 months for the high-risk group [5,6,38]. A further resolution of the mortality risk would be of daily relevance. Therefore, liver stiffness might be useful in identifying high-risk patients requiring intensive monitoring and without the benefit of disease-modifying treatments. It would allow for the more efficient use of resources by implementing it in the clinical pathway after diagnosis against the backdrop of improved awareness and increasing numbers of newly diagnosed patients [39,40].

Liver vein dilation was chosen as the primary surrogate for congestion in our analysis, as it was a clearly defined measure with low investigator dependency on the one hand. On the other hand, a correlation analysis regarding liver stiffness showed that there were significant correlations between liver stiffness and echo-based assessed congestion markers such as tr-v_max_, liver vein dilation and right atrial volume, whereas the number of diuretics, diuretic equivalence dose, neck vein dilation, oedema and the combined scores of clinical congestion signs failed to reach significance. Of course, the effect of the small number of subjects can be discussed in this context, especially as the assessment of clinical parameters may be more variable than the standardised assessment of defined echocardiographic parameters by experienced echocardiographers. However, clinical experience shows that amyloidosis patients, as typical examples of restrictive cardiomyopathy, are often hypervolemic even in the absence of classic signs of heart failure such as oedema. It remains to be evaluated which is the best parameter for detecting hypervolemia in cardiac amyloidosis and whether there may also be subtype-specific differences.

The limitations of our study are the non-availability of a liver biopsy for histological correlation, which would have been desirable as the gold standard for the detection and staging of fibrosis and a possible inflammatory component. Because of this particular bleeding risk, there is a complete lack of previous histological data up to now and the congestion-adjusted version of the FIB-4 appears to be an acceptable surrogate parameter with careful risk–benefit consideration. As the analyses refer to the data set of a registry study, a dedicated control group for heart failure is not available. We addressed this issue by selecting a mixed control group consisting of patients with excluded cardiac amyloidosis, including patients with signs of heart failure (approximately 40% of NYHA stage II–III patients in the control group). The post-cluster LASSO was additionally performed with the subgroup of localised amyloidosis, which is considered to be cardiac-healthy patients, to get an idea of possible differences.

Due to the small number of cases, the results have to be considered exploratory and require further confirmation and validation. The small case number was addressed by using a panel data set and a cross-subtype analysis with subsequent consideration of the respective group due to the different pathomechanisms. At the same time, however, the cross-subtype approach allows for better generalisability.

Future investigations regarding the validation of the results in an independent cohort, as well as the comparison with other cardiac diseases, but also the development of stratification algorithms with liver stiffness in combination with other parameters, appear promising. A precise characterisation of the metabolic limitations in the various subtypes of amyloidosis may allow for the identification of a specific pattern in the long term, which can be used in dedicated centres for the non-invasive diagnosis of hepatic amyloidosis.

## 5. Conclusions

Clinical findings and in daily practice established liver function tests such as AP fail to differentiate between primary and secondary liver affection in CA. Liver stiffness might be a promising clinical tool for combined imaging of liver fibrosis, impaired cardiac function, hypervolemia and congestion in a time-sparing and easy-to-use manner. Reduced microsomal liver function in ATTR-CA seems to be related to hepatic congestion, whereas lower values in AL-CA may be explained by changed hepatic blood flow. Liver stiffness is a predictor of all-cause mortality and might be a promising parameter for risk stratification in cardiac amyloidosis, but further investigation and confirmation are required.

## Figures and Tables

**Table 1:** Basic characterisation of the cohort.

		ATTR-CA				AL-CA				Control				ATTR-CA vs. Control		AL-CA vs. Control		ATTR-CA vs. AL-CA	
n patients		26				17				31									
n observations		60				52				65									
		*n*	*mean*		*SD*	*n*	*mean*		*SD*	*n*	*mean*		*SD*	*z*	*p value*	*z*	*p value*	*z*	*p value*
**age [years]**		26	74.9	±	7.2	17	64.0	±	8.1	31	63.1	±	11.6	4.6781	***	0.3162	n.s.	4.500	***
**sex**	*male*	26	84.6%			17	41.2%			31	54.8%								
	*female*	26	15.4%			17	58.8%			31	45.2%								
**ECOG**		26	0.5	±	0.5	16	0.5	±	0.7	30	0.2	±	0.5	2.0418	*	1.5588	n.s.	−0.061	n.s.
**cardiac comorbidity**		26	76.9%	±	43.0%	17	52.9%	±	51.4%	31	45.2%	±	50.6%	2.5631	*	0.5040	n.s.	1.593	n.s.
**hepatic comorbidity**		26	19.2%	±	40.2%	16	12.5%	±	34.2%	31	22.6%	±	42.5%	−0.3053	n.s.	−0.8801	n.s.	0.579	n.s.
**number of involved organs by amyloidosis**		26	1.6	±	0.5	17	2.0	±	1.3	31	1.2	±	0.5	2.7619	**	2.4877	*	−1.303	n.s.
**severity of cardiac amyloidosis ^#^**																			
	*stage I*	15	58%			1	6%												
	*stage II*	8	31%			5	29%												
	*stage III*	3	12%			11	65%												
**NYHA**	*I*	6	23.1%			6	28.6%			18	58.1%								
	*II*	8	30.8%			8	38.1%			9	29.0%								
	*III*	12	46.2%			6	28.6%			4	12.9%								
	*IV*	0	0.0%			1	4.8%			0	0.0%								
**rhythm**	*sinus rhythm*	26	53.8%	±	50.8%	17	82.4%	±	39.3%	31	90.3%	±	30.1%	−3.2173	**	−0.7276	n.s.	−2.067	*
	*atrial fibrillation*	26	38.5%	±	49.6%	17	17.6%	±	39.3%	31	9.7%	±	30.1%	2.5869	**	0.7276	n.s.	1.528	n.s.
	*pacemaker rhythm*	26	7.7%	±	27.2%	17	0.0%	±	0.0%	31	0.0%	±	0.0%	1.4434	n.s.	N/A	n.s.	1.443	n.s.
**NT-proBNP [pg/mL]**		26	2782.6	±	2290.8	17	9601.3	±	13,988.5	31	320.8	±	552.4	5.3507	***	2.7343	**	−1.992	*
**troponin [pg/mL]**		26	49.1	±	24.9	17	99.9	±	88.9	31	11.5	±	7.8	7.3875	***	4.0893	***	−2.299	*
**eGFR_MDRD_ [mL/min]**		26	64.8	±	18.9	17	58.9	±	29.8	31	75.8	±	19.3	−2.1583	*	−2.1125	*	0.735	n.s.
**septum [mm]**		26	16.7	±	3.7	17	13.5	±	2.6	31	10.0	±	1.4	8.6865	***	5.0339	***	3.310	***
**posterior wall [mm]**		26	13.9	±	2.6	17	11.8	±	2.1	31	9.2	±	1.5	8.2203	***	4.4663	***	2.956	**
**LVEF [%]**		26	54.3	±	12.4	17	62.1	±	11.4	31	63.5	±	5.5	−3.5077	***	−0.4557	n.s.	−2.139	*
**cardiac output [L/min]**		23	4.3	±	1.5	13	6.2	±	2.7	29	5.4	±	1.2	−2.9021	***	1.0297		−2.338	**
**stroke volume [mL]**		23	66.1	±	21.5	15	78.6	±	38.0	30	82.8	±	22.4	−2.7577	**	−0.3982	n.s.	−1.158	n.s.
**GLPS [%]**		24	−11.3	±	3.3	16	−13.4	±	4.1	30	−18.3	±	2.3	8.7357	***	4.5080	***	1.641	n.s.
**apical sparing**		26	100.0%	±	100.0%	17	88.2%	±	66.8%	31	45.2%	±	49.4%	2.5475	*	2.3320	n.s.	0.463	n.s.
**E/A**		13	1.9	±	1.4	13	2.2	±	1.0	27	1.0	±	0.3	2.2190	*	4.0794	***	−0.677	n.s.
**E/E’**		22	15.5	±	6.5	16	15.9	±	7.5	30	8.6	±	2.1	4.8143	***	3.8022	***	−0.156	n.s.
**tr-v_max_ [m/s]**		18	3.0	±	0.4	15	2.8	±	0.3	19	2.4	±	0.3	3.9130	***	3.4562	***	0.916	n.s.
	*% normal*	8	44%			5	33%			17	89%								
	*% pathologic* (>2.8)	10	56%			10	67%			2	11%								
**diastolic dysfunction**		26	73.1%	±	45.2%	17	76.5%	±	43.7%	31	12.9%	±	34.1%	5.5832	***	5.1917	***	−0.245	n.s.
**acute heart failure according to ESC criteria**		26	69.2%	±	47.1%	17	76.5%	±	43.7%	31	67.7%	±	47.5%	0.1184	n.s.	0.6412	n.s.	−0.515	n.s.
**walking distance in 6 min walking test [m]**		23	367.3	±	84.9	14	374.3	±	112.7	23	402.6	±	111.1	−1.2095	n.s.	−0.7453	n.s.	−0.199	
**total bilirubin [mg/dL]**		26	0.8	±	0.3	17	0.6	±	0.3	31	0.5	±	0.2	5.3905	***	1.8334	*p* < 0.1	1.906	*p* < 0.1
**AP [U/L]**		26	85.8	±	41.0	17	74.8	±	22.7	31	69.9	±	17.3	1.8448	*p* < 0.1	0.7626	n.s.	1.136	n.s.
**GGT [U/L]**		26	94.8	±	92.4	17	47.6	±	44.3	31	30.6	±	14.8	3.5049	***	1.5370	n.s.	2.238	*
**AST [U/L]**		26	34.9	±	11.7	17	29.8	±	15.2	31	25.0	±	7.8	3.6853	***	1.2144	n.s.	1.179	n.s.
**ALT [U/L]**		26	30.3	±	13.8	17	38.4	±	57.7	31	26.4	±	13.8	1.0577	n.s.	0.8492	n.s.	−0.575	n.s.
**GLDH [U/L]**		25	4.8	±	3.5	17	5.1	±	6.1	31	4.1	±	7.1	0.4327	n.s.	0.4931	n.s.	−0.204	n.s.
**cholinesterase [U/L]**		25	6944.7	±	1615.3	17	6287.8	±	2098.8	31	8352.9	±	1850.7	−3.0381	**	−3.3968	***	1.090	n.s.
**serum albumin [g/dL]**		26	4.5	±	0.2	17	3.9	±	0.7	31	4.3	±	0.8	1.2566	n.s.	−1.6510	*p* < 0.1	3.209	**
**liver vein dilatation (%)**		26	11.5%			15	6.6%			30	0.0%			5.0990	*	0.9904	n.s.	0.696	n.s.
**FIB−4**		26	2.77	±	0.96	17	2.34	±	1.84	31	1.37	±	0.60	6.4743	***	2.1131	**	0.896	n.s.
**PDR_peak_ [%]**		22	25.9	±	7.1	13	24.5	±	5.6	27	31.3	±	15.2	−1.6334	n.s.	−2.0640	*	0.677	n.s.
**stiffness [kPa]**		20	7.9	±	4.5	11	5.0	±	2.0	24	5.5	±	3.3	1.9779	*	−0.6534	n.s.	2.533	*
**IQR med [%]**		20	24.6	±	14.0	10	16.3	±	3.8	21	22.4	±	8.9	0.5752	n.s.	−2.6804	**	2.453	*

Baseline characterisation of the analysed AmyKoS subgroups with special focus on cardiac and hepatic parameters. Differences between subgroups were analysed by *z*-test. * *p* < 0.05, ** *p* < 0.01, *** *p* < 0.001; n.s. = not significant; ^#^ Gillmore stage for ATTR-CA; modified Mayo stage for AL-CA.

**Table 2:** Effects of congestion on PDR_peak_ and FIB-4.

NT-proBNP as congestion surrogate			
		PDR_peak_ HR (95% CI)	FIB-4 HR (95% CI)
n observations		148	177
constant		29.805 *** [25.646; 33.964]	1.451 *** [1.218; 1.684]
cardiac manifestation	ATTR-CA	−0.737 [−6.593; 5.118]	1.352 *** [0.520; 2.185]
	AL-CA	−5.849 ** [−10.460; −1.238]	0.600 * [−0.053; 1.253]
NT-proBNP ^#^	overall	−1.726 ** [−3.445; −0.006]	0.036 [−0.034; 0.107]
	ATTR-CA	0.787 [−1.216; 2.789]	−0.039 [−0.189; 0.112]
	AL-CA	1.652 * [−0.070; 3.374]	−0.020 [−0.096; 0.057]
**liver vein dilation as congestion surrogate**			
n observations		143	172
constant		28.921 *** [25.182; 32.660]	1.478 *** [1.255; 1.700]
cardiac manifestation	ATTR-CA	−1.429 [−6.311; 3.453]	1.326 *** [0.774; 1.878]
	AL-CA	−5.130 ** [−9.533; −0.726]	0.700 ** [0.034; 1.367]
dilated liver veins	overall	−2.240 * [−4.738; 0.258]	−0.168 [−1.242; 0.907]
	ATTR-CA	−6.021 ** [−11.390; −0.653]	0.304 [−0.893; 1.501]
	AL-CA	^##^	^##^

The 95% confidence intervals (95% CI) in brackets; * *p* < 0.1, ** *p* < 0.05, *** *p* < 0.01; ^#^ in 1000 pg/mL; ^##^ dropped due to multicollinearity. A multivariate regression analysis based on the panel data set was performed to evaluate the effect of cardiac congestion on PDR_peak_ and FIB-4. NT-proBNP and dilated liver veins were chosen as surrogates for congestion: PDR_peak_ was significantly reduced in AL-CA (column 1: b_NT-proBNP_ = −5.849, *p* < 0.05; b_liver vein dilation_ = −5.130, *p* < 0.05), but not in ATTR-CA (column 1: b_NT-proBNP_ = −0.737, *p* > 0.1; b_liver vein dilation_ = −1.429, *p* > 0.1). NT-proBNP was significantly negatively correlated with PDR_peak_ overall (column 1: b_overall_ = −1.726, *p* < 0.05). The effect of NT-proBNP on PDR_peak_ was comparable to the overall effect for ATTR-CA (column 1: b_ATTR-CA_ = 0.787, *p* > 0.1), but in AL-CA it was negligible because both the overall (column 1: b_overall_ = −1.726, *p* < 0.05) and the group-specific effect (column 1: b_AL-CA_ = 1.652, *p* < 0.1) were significant and their sum was close to zero. Liver vein dilatation was associated with a significant decrease in PDR_peak_ overall (column 1: b_overall_ = −2.240 *p* < 0.1), which was more pronounced and significant in ATTR-CA in the subtype-specific analysis (column 1: b_ATTR-CA_ = −6.021, *p* < 0.05)). This effect could not be calculated in the AL-CA subgroup due to multicollinearity indicating that the effect is comparable to the baseline effect. Both AL-CA (column 2: b_NT-proBNP_ = 0.600, *p* < 0.1; b_liver vein dilation_ = −0.700, *p* < 0.05) and ATTR-CA (column 2: b_NT-proBNP_ = 1.352, *p* < 0.01; b_liver vein dilation_ = 1.326, *p* < 0.01) showed a significant baseline effect on FIB-4, but there was no significant effect on FIB-4 by NT-proBNP and liver vein dilatation.

**Table 3:** Results of post-cluster LASSO.

		Model 1	Model 2
n observations		76	101
n patients		35	52
**Core Variables**		**Log Liver Stiffness**	**Log Liver Stiffness**
septum thickness	control	−0.136 ** [−0.255; −0.016]	−0.032 [−0.149; 0.085]
	ATTR-CA	0.021 ** [0.002; 0.040]	0.023 ** [0.002; 0.044]
	AL-CA	0.055 *** [0.016; 0.094]	0.077 *** [0.041; 0.113]
NT-proBNP	control	1.722 ** [0.165; 3.279]	0.036 [−0.032; 0.105]
	ATTR-CA	0.069 *** [0.028; 0.111]	0.070 *** [0.021; 0.118]
	AL-CA	0.002 [−0.002; 0.007]	0.003 [−0.001; 0.008]
PDR_peak_	control	−0.003 [−0.018; 0.012]	−0.014 [−0.033; 0.004]
	ATTR-CA	−0.026 *** [−0.041; −0.011]	−0.025 *** [−0.040; −0.010]
	AL-CA	−0.031 *** [−0.048; −0.015]	−0.034 *** [−0.051; −0.017]
dilated liver veins	control	^#^	^#^
	ATTR-CA	0.376 ** [0.064; 0.688]	0.438 *** [0.117; 0.759]
	AL-CA	0.066 [−0.229; 0.362]	0.020 [−0.262; 0.302]
FIB−4_clean_	control	0.132 [−0.230; 0.494]	0.179 [−0.205; 0.564]
	ATTR-CA	0.144 *** [0.043; 0.245]	0.153 *** [0.046; 0.260]
	AL-CA	0.011 [−0.064; 0.087]	0.024 [−0.051; 0.099]
LASSO-selected controls		AL-CA, ATTR-CA, AP, TAPSE	AL-CA, ATTR-CA, AP
constant		3.215 *** [1.544; 4.885]	2.359 *** [0.994; 3.725]

The 95% confidence intervals are in brackets; * *p* < 0.1, ** *p* < 0.05, *** *p* < 0.01. Each of the two columns represents a multivariate regression of the log normalized liver stiffness on the core variables and the high-dimensional controls selected by post-cluster LASSO. The core variables of interest were septum thickness, NT-proBNP, PDRpeak and dilation of liver veins within both models pre-selected based on Figure 1. Coefficients for liver vein dilation (^#^) were not identified, as there was no patient with dilated hepatic veins among the control group. The control group used in model 1 included only the subset of control patients with localised amyloidosis without cardiac impairment, whereas model 2 referred to the entire control group with a high percentage of patients suffering from other cardiac disorders.

**Table 4:** COX analysis.

COX Analysis	1	2
	HR [95% CI]	HR [95% CI]
liver stiffness	1.744 ** [1.136; 2.678]	
NT-proBNP (in 1000)	1.025 * [0.997; 1.054]	
hs-TNT	1.120 ** [1.027; 1.222]	
PDR_peak_	1.305 *** [1.079; 1.579]	
dilated liver veins		14.101 [0.244; 813.970]
septum thickness		1.062 [0.876; 1.288]
FIB-4_clean_		2.798 [0.365; 21.425]
n observations	105	105
Pseudo R^2^	0.642	0.272

Exponentiated coefficients; 95% confidence intervals in brackets. * *p* < 0.1, ** *p* < 0.05, *** *p* < 0.01. Cox proportional hazard regression (Cox survival regression) was used to evaluate the ability of the parameters used in the initial LASSO regression model (stiffness, septum thickness, NT-proBNP, liver vein dilatation, PDR_peak_) to predict mortality risk. High-sensitive troponin, NT-proBNP, PDR_peak_ and liver stiffness were able to predict prognosis (column 1) in contrast to septum thickness, dilated liver veins and FIB-4_clean_ (column 2).

## Data Availability

Data cannot be shared in public due to privacy regulations and patient consent.

## Supplementary Materials

The following supporting information can be downloaded at: https://www.mdpi.com/article/10.3390/jcm13051440/s1, Table S1: Overview of diagnostic investigations to assess liver affection in cardiac amyloidosis; Table S2: Additional information on clinical signs of congestion/hypervolemia and medication; Table S3: Comparison of PDRpeak among different patient collectives; Table S4: Surrogate parameters for the main influencing factors and the rationale of their choice; Table S5: Results of post-cluster LASSO with addition of tr-vmax as surrogate of acutal volume status.

## Abbreviations

AL amyloidosis	systemic light chain amyloidosis
AL-CA	cardiac light chain amyloidosis
ALF	acute liver failure
ALT	alanine aminotransferase (=GPT)
AP	alkaline phosphatase
APRI	AST to platelet ratio index
AST	aspartate aminotransferase (=GOT)
ATTR amyloidosis	transthyretin amyloidosis
ATTR-CA	cardiac transthyretin amyloidosis
AUC	area under the curve
CA	cardiac amyloidosis
COX survival regression	Cox proportional hazard regression
CYP1A2	cytochrome P450 1A2
ECOG Performance Status	Eastern Cooperative Oncology Group Performance Status
eGFR	estimated glomerular filtration rate
FIB-4 (score)	fibrosis-4 (score)
FIB-4_clean_	fibrosis-4 (score) cleaned for its statistical component related to congestion by regressing the score on NT-proBNP and liver vein congestion
GGT	gamma-glutamyl transferase
GLDH	glutamate dehydrogenase
GLPS	global longitudinal strain
GOT	aspartate aminotransferase (AST)
GPT	alanine aminotransferase (ALT)
HCV	hepatitis C virus
HFpEF	heart failure with preserved ejection fraction
IQR	interquartile range
IQR med	IQR/median
kPA	kilopascal
LASSO	least absolute shrinkage and selection operator regression
LVEF	left ventricular ejection fraction
LSS	Ludwig’s staging system
MAPK pathway	mitogen-activated protein kinase pathway
MBT	^13^C-methacetin breath test
ML	machine learn
NT-proBNP	N-terminal pro-brain natriuretic peptide
NYHA	New York Heart Association
PBC	primary biliary cholangitis
PDRpeak	maximal percentage of dose rate
PDR_20_	percentage of dose rate at 20 min
PK model	pharmacokinetic model
posterior wall	left ventricular posterior wall
septum	interventricular septum
TAPSE	tricuspid annular plane systolic excursion
tr-v_max_	maximal tricuspid regurgitation velocity
ULN	upper limit of normal
VTCE	vibration-controlled transient elastography
6MWT	6 min walk test
