# Supplementary material for: Cardio-Hepatic Interaction in Cardiac Amyloidosis
Source: J Clin Med. 2024 Mar 1;13(5):1440. doi: 10.3390/jcm13051440 (PMC10932330; doi:10.3390/jcm13051440)
Supplement: Supplementary file 1 [file jcm-13-01440-s001.zip › jcm-2821286-supplementary.pdf]

Journal of
Clinical Medicine
MDPI

Table S1. *Overview of diagnostic investigations to assess liver affection in cardiac amyloidosis*

Method	*Mode of assessment*	*explanation*
AST	laboratory value (blood)	liver-specific enzyme acting as an indicator of liver cell damage (mainly cytoplasmatic localisation)
ALT	laboratory value (blood)	non-liver-specific enzyme serving as an indicator of liver cell damage (mainly mitochondrial localisation)
Alkaline phosphatase (AP)	laboratory value (blood)	application as a cholestasis parameter in clinical practice;<br>specifically in amyloidosis, AP is used as a diagnostic criterion for a hepatic AL amyloidosis manifestation according to Gertz et al.[11]
GLDH	laboratory value (blood)	liver-specific, purely mitochondrial enzyme acting as an indicator of (severe) liver cell damage
cholinesterase	laboratory value (blood)	indicator of liver synthesis performance e.g. reduced levels can be found in patients with liver cirrhosis
FIB-4 score	Lab-based score	The FIB-4 score can be calculated from AST, ALT, platelet count and age according to the formula in the section “methods”[26-28]. It can be used for the non-invasive assessment of liver fibrosis risk in non-alcoholic fatty liver disease. Values greater than 3.25 indicate a high probability of advanced fibrosis[26].
VTCE	ultrasound-based assessment of liver stiffness	Vibration-controlled transient elastography (VTCE) is an ultrasound-based procedure. A probe placed above the liver is used to emit a single mechanical impulse and then the pulse wave velocity is measured in the liver tissue. The velocity of the pulse propagation is proportional to the stiffness of the liver. Standard values range from 3 to 7.5 kPa. Sensitivity and specificity depend on the degree of fibrosis[29]. False high values can be measured in cases of increased arterial perfusion after food intake, venous congestion/right heart failure, cholestasis, infiltrative liver disease and acute hepatitis[30-34].
^{13}C-methacetin breath test [35]	breath test	During the ^{13}C-methacetin breath test, patients are administered ^{13}C-labelled methacetin on fasting [35]. This is metabolised in the liver and the patients then breathe out ^{13}C-labelled CO_2.<br>The $^{13}C/^{12}C$ ratio in breath can be measured over 60 minutes in 10-minute intervals by nondispersive isotope selective infrared spectroscopy. Based on this, the maximal percentage dose rates (PDRmax [%/h]) can be calculated which is a measure for microsomal liver function.<br>By choice of the ^{13}C-labeled substrate different compartiments of liver function can be evaluated e.g. microsomal function by ^{13}C-methacetin, mitochondrial function by ^{13}C-methionin and cytosolic function by ^{13}C-galactose.

Table S2. *Additional information on clinical signs of congestion/hypervolemia and medication*

	ATTR-CA				AL-CA				control			
	n	*mean*		*SD*	*n*	*mean*		*SD*	*n*	*mean*		*SD*
**clinical congestion score**[#]	20	1.9	±	1.2	12	2.2	±	1,6	27	1,4	±	0,6
**signs of congestion/heart failure**												
0	23	4.3%	(1)		14	14.3%	(2)		27	3.7%	(1)	
1	23	34.8%	(8)		14	21.4%	(3)		27	51.9%	(14)	
2	23	30.4%	(7)		14	14.3%	(2)		27	40.7%	(11)	
≥ 3	23	30.4%	(7)		14	50.0%	(7)		27	3.7%	(1)	
**oedema**	26	30.8%	(8)		17	47.1%	(8)		30	23.3%	(7)	
**dilated neck vein**	24	41.7%	(10)		16	60.0%	(9)		29	17.2%	(5)	
**rales**	26	3.8%	(1)		17	11.8%	(2)		30	0.0%	(0)	
**orthopnoea**	21	9.5%	(2)		15	13.3%	(2)		30	3.3%	(1)	
**cough**	26	23.1%	(6)		15	13.3%	(2)		30	10.0%	(3)	
**nykturia**	26	92.3%	(24)		16	62.5%	(10)		31	90.3%	(28)	
**diuretics**					17	58.9%	(10)					
**number of diuretics**	26	1.0	±	0.8	17	0.94	±	0.82	31	0.29	±	0.53
**loop diuretics**[##]	26	69.2%	(18)		17	58.8%	(10)		31	19.4%	(6)	
**thiazide diuretics**	26	7.7%	(2)		17	23.5%	(4)		31	9.7%	(3)	
**mineralocorticoid receptor antagonists**	26	23.1%	(6)		17	11.8%	(2)		31	0.0%	(0)	
**ACEI**	26	34.6%	(9)		17	23.5%	(4)		31	6.5%	(2)	
**angiotensin II receptor antagonists**	26	15.4%	(4)		17	11.7%	(2)		31	38.7%	(12)	
**betablocker**	26	65.4%	(17)		17	31.6%	(6)		31	35.5%	(11)	
**calcium antagonists**	26	15.4%	(4)		17	0%	(0)		31	9.7%	(3)	
**anticoagulation**	26	53.8%	(14)		17	17.6%	(3)		31	12.9%	(4)	
**other cardiac medication****	26	3.8%	(1)		17	17.6%	(3)		31	6.5%	(2)	
**causal treatment**												
**tafamidis**	26	11.5%	(3)		17	0.0%	(0)		31	3.2%***	(1)	
**EGCG**	26	19.2%	(5)		17	17.6%	(3)		31	6.4%***	(2)	
**plasma cell directed treatment**	26	0.0%	(0)		17	47.1%*	(8)		31	0.0%	(0)	

[#]sum of clinical signs of congestion: edema, dilated neck veins, orthopnea, cough, nykturia

[##] median daily dosage of diuretics among those treated with diuretics was equivalent to 17.5 (10.0;20.0) mg torasemide among ATTR-CA, 30.0 (16.25;55.00) mg among AL-CA and 12.5 (9.38;16.25) mg among controls, respectively.

* 8 patients under ongoing treatment at first evaluation, 7 patients with treatment in the past, 2 treatment naïve patients patients before treatment initiation

** mainly antiarrhythmic medication

*** the control group included two patients with extracardiac evidence of ATTRwt amyloid in the bladder and as incidental finding after carpal tunnel operation without cardiac involvement and one ATTRv patient with isolated neurological involvement

Table S3: *Comparison of PDR_{peak} among different patient collectives*

			PDRpeak		ATTR-CA		AL-CA		strict control		main control		broad control	
**Data source**	**Collective**	***mean***	***sd***	***n***	***z-test***	***p value***	***z-test***	***p value***	***z-test***	***p value***	***z-test***	***p value***	***z-test***	***p value***
analysed data set	ATTR-CA	25,92	7,07	22		N/A	-0,675	n.s.	0,697	n.s.	1,098	n.s.	1,292	n.s.
	AL-CA	24,45	5,63	13	0,675	n.s.		N/A	1,035	n.s.	1,563	n.s.	1,897	n.s.
	strict control	28,91	9,83	6	-0,697	n.s.	-1,035	n.s.		N/A	0,077	n.s.	0,000	n.s.
	main control	29,28	10,65	16	-1,098	n.s.	-1,563	n.s.	-0,077	n.s.		N/A	-0,117	n.s.
	Broad control	28,91	8,94	26	-1,292	n.s.	-1,897	p<0.1	0,000	n.s.	0,117	n.s.		N/A
Ciccocioppo et al. 2002	elderly (mean age 79.8 years, SD 7.9 years, range 67-100 years)	30,66	9,20	29	-2,080	*	-2,682	**	-0,401	n.s.	-0,436	n.s.	-0,716	n.s.
	adults (mean age 40.6 years, SD 12.3 years, range 44-62 years)	38,33	6,05	28	-6,559	***	-7,172	***	-2,257	*	-3,123	**	-4,501	***
Pfaffenbach et al. 1998	healthy	43,07	14,60	20	-4,769	***	-5,144	***	-2,737	**	-3,273	**	-3,821	***
Lalazar et al. 2007	HCV low inflammation	38,30	15,73	32	-3,912	***	-4,340	***	-1,922	p<0.1	-2,341	**	-2,855	**
	HCV high inflammation	28,59	11,20	68	-1,315	n.s.	-1,998	*	0,076	n.s.	0,231	n.s.	0,144	n.s.
	HCV non-significant fibrosis (HAIf ≤2)	36,84	11,37	50	-4,955	***	-5,528	***	-1,835	p<0.1	-2,431	*	-3,335	***
	HCV significant fibrosis (HAIf >2)	26,55	13,66	50	-0,256	n.s.	-0,843	n.s.	0,531	n.s.	0,831	n.s.	0,905	n.s.
	healthy (mean age 40.7, sd 12.6, range 18-75)	35,31	8,94	100	-5,357	***	-6,035	***	-1,557	n.s.	-2,147	*	-3,253	**
	HCV total (mean age 46.3, sd 13.6, range 19-76)	31,70	13,53	100	-2,853	**	-3,508	***	-0,659	n.s.	-0,810	n.s.	-1,261	n.s.
Goetze 2020	HCV (Swiss cohort, mean age 46.2 years, SD 11.3 years, range 20-74 years)	28,18	12,92	224	-1,301	n.s.	-2,089	*	0,178	n.s.	0,393	n.s.	0,373	n.s.
	Ishak hepatitis 0	30,94	12,57	92	-2,513	*	-3,182	**	-0,481	n.s.	-0,559	n.s.	-0,928	n.s.
	Ishak hepatitis 1	27,83	12,16	96	-0,979	n.s.	-1,693	p<0.1	0,257	n.s.	0,493	n.s.	0,501	n.s.
	Ishak hepatitis 2	24,15	11,00	6	0,373	n.s.	0,063	n.s.	0,790	n.s.	0,982	n.s.	0,986	n.s.
	Ishak portal inflammation 0	30,46	13,63	6	-0,788	n.s.	-1,040	n.s.	-0,226	n.s.	-0,192	n.s.	-0,266	n.s.
	Ishak portal inflammation 1	29,76	12,14	122	-2,058	*	-2,779	**	-0,204	n.s.	-0,166	n.s.	-0,412	n.s.
	Ishak portal inflammation 2	27,81	13,18	61	-0,837	n.s.	-1,462	n.s.	0,252	n.s.	0,465	n.s.	0,450	n.s.
	Ishak portal inflammation 3	30,58	8,51	5	-1,139	n.s.	-1,489	n.s.	-0,302	n.s.	-0,280	n.s.	-0,399	n.s.
	Ishak fibrosis 0	35,01	14,86	16	-2,266	*	-2,618	**	-1,115	n.s.	-1,253	n.s.	-1,485	n.s.
	Ishak fibrosis 1	30,57	9,99	61	-2,351	*	-3,029	**	-0,393	n.s.	-0,436	n.s.	-0,764	n.s.
	Ishak fibrosis 2	31,71	13,27	39	-2,224	*	-2,753	**	-0,617	n.s.	-0,714	n.s.	-1,018	n.s.
	Ishak fibrosis 3	31,25	12,78	37	-2,060	*	-2,595	**	-0,516	n.s.	-0,579	n.s.	-0,854	n.s.
	Ishak fibrosis 4	26,86	16,14	7	-0,149	n.s.	-0,382	n.s.	0,281	n.s.	0,364	n.s.	0,323	n.s.
	Ishak fibrosis 5	21,16	7,56	15	1,931	p<0.1	1,319	n.s.	1,737	p<0.1	2,460	*	2,954	**
	Ishak fibrosis 6	18,19	7,21	18	3,403	***	2,716	**	2,460	*	3,511	***	4,390	***
	Metavir grading (inflammation) 0	31,03	14,11	47	-2,001	*	-2,543	*	-0,469	n.s.	-0,518	n.s.	-0,783	n.s.
	Metavir grading (inflammation) 1	28,62	10,96	130	-1,508	n.s.	-2,271	*	0,071	n.s.	0,234	n.s.	0,146	n.s.
	Metavir grading (inflammation) 2	28,51	17,28	17	-0,582	n.s.	-0,907	n.s.	0,069	n.s.	0,155	n.s.	0,088	n.s.
	Metavir staging (fibrosis) 0	35,47	14,52	17	-2,493	*	-2,860	**	-1,229	n.s.	-1,402	n.s.	-1,668	n.s.
	Metavir staging (fibrosis) 1	30,89	11,32	99	-2,633	**	-3,334	***	-0,476	n.s.	-0,557	n.s.	-0,950	n.s.
	Metavir staging (fibrosis) 2	31,25	12,78	37	-2,060	*	-2,595	**	-0,516	n.s.	-0,579	n.s.	-0,854	n.s.
	Metavir staging (fibrosis) 3	26,86	16,14	7	-0,149	n.s.	-0,382	n.s.	0,281	n.s.	0,364	n.s.	0,323	n.s.
	Metavir staging (fibrosis) 4	18,92	7,74	35	3,509	***	2,720	**	2,368	*	3,493	***	4,567	***
Kochel-Jankowska 2013	healthy (age 55.4 years, range 33-70 years, only female)	34,60	1,90	16	-5,491	***	-6,219	***	-1,408	n.s.	-1,967	n.s.	-3,134	n.s.
	Primary biliary cholangitis - LSS I	34,10	3,20	6	-4,100	***	-4,739	***	-1,230	n.s.	-1,625	n.s.	-2,375	*
	Primary biliary cholangitis - LSS II	31,00	3,00	9	-2,808	p<0.1	-3,531	***	-0,505	n.s.	-0,605	n.s.	-1,036	n.s.
	Primary biliary cholangitis - LSS III	28,40	3,40	9	-1,315	n.s.	-2,045	*	0,122	n.s.	0,304	n.s.	0,243	n.s.
	Primary biliary cholangitis - LSS IV	15,10	3,10	8	5,805	***	4,905	***	3,320	***	4,924	***	6,678	***
Fontana et al.	ALF 21d transplant free survivor (measured day 1)	10,20	7,80	23	7,088	***	6,324	***	4,321	***	6,115	***	7,823	***
	ALF 21d non-survivor (measured day 1)	1,90	0,60	12	15,828	***	14,363	***	6,724	***	10,260	***	15,330	***
	ALF 21d transplant free survivor (measured day 1 or 2)	9,10	6,80	35	8,872	***	7,921	***	4,746	***	6,958	***	9,448	***
	ALF 21d non-survivor (measured day 1 or 2)	2,30	0,90	21	15,536	***	14,083	***	6,623	***	10,104	***	15,082	***
Vranova et al. 2012	alcohol-induced cirrhosis CHILD A (PDR20)	23,27	10,68	10	0,716	n.s.	0,318	n.s.	1,075	n.s.	1,397	n.s.	1,482	n.s.
	alcohol-induced cirrhosis CHILD B (PDR20)	12,93	12,54	28	4,625	***	4,061	***	3,429	***	4,587	***	5,420	***
	alcohol-induced cirrhosis CHILD C (PDR60)	5,01	3,63	14	11,663	***	10,581	***	5,789	***	8,563	***	11,926	***
	alcohol-induced cirrhosis overall (PDR20)	12,53	12,38	52	5,860	***	5,139	***	3,753	***	5,287	***	6,674	***
	controls (mean age 58.8 years, SD 12.6 years, range 38-90 years, female	32,48	9,31	37	-3,053	**	-3,671	***	-0,831	n.s.	-1,042	n.s.	-1,535	n.s.

Potential reference groups were identified via PubMed search using the general keyword "methacetin breath test". Minimum requirements for the use as a reference group were the clear definition of the reported collective by a single underlying disorder and the specification of mean, standard deviation and total number of patients analysed for PDR_{peak} to enable comparison with the CA patients.

Table S4. *Surrogate parameters for the main influencing factors and the rationale of their choice.*

Main influencing factor	*Surrogate parameter*	*Rationale*
severity of CA	septum thickness	well available, albeit non-specific parameter expressing degree of hypertrophy and amyloid burden, established diagnosis criterion according to Gertz et al. 2005
severity of CA/congestion	NT-proBNP	combined parameter for severity of cardiac involvement and cardiac congestion, known to be highly prognostic relevant in cardiac amyloidosis across subtypes and even in patients without cardiac involvement, depicts direct cardiotoxicity of circulating free light chains in AL-CA
severity of CA	high-sensitive troponin	well-established parameter expressing severity of cardiac involvement, known to be highly prognostic relevant in cardiac amyloidosis across subtypes
congestion	dilatation of liver veins	direct and well available parameter of cardiac congestion
liver cell damage	PDRpeak	direct marker of microsomal liver function; additionally decreased in AL-CA because of altered blood flow
liver cell damage	FIB-4/$FIB\text{-}4_{clean}$	indicator of liver fibrosis cleaned for its congestion-related component
inflammation	C-reactive protein	general marker of inflammation

Table S5. *Results of post-cluster LASSO with addition of tr-v_{max} as surrogate of acutal volume status*

		model 1	model 2
n observations		57	
**core variables**		**log liver stiffness**	**log liver stiffness**
septum thickness	Control	-1.081 [-3.734,1.573]	0.060 [-0.034,0.154]
	ATTR-CA	0.017 [-0.017,0.050]	0.019 [-0.015,0.054]
	AL-CA	0.038 [-0.035,0.110]	0.079*** [0.029,0.130]
NT-proBNP	Control	##	-0.195*** [-0.330,-0.061]
	ATTR-CA	0.050 [-0.041,0.141]	0.087* [-0.003,0.178]
	AL-CA	0.005* [-0.001,0.011]	0.005** [0.000,0.011]
PDR_{peak}	Control	0.081[-0.166,0.329]	-0.013 [-0.031,0.005]
	ATTR-CA	-0.040*** [-0.056,-0.025]	-0.040*** [-0.057,-0.023]
	AL-CA	-0.025** [-0.050,-0.000]	-0.031*** [-0.053,-0.010]
dilated liver veins	Control	#	#
	ATTR-CA	0.254 [-0.071,0.579]	0.348** [0.012,0.684]
	AL-CA	0.072 [-0.230,0.374]	0.012 [-0.236,0.261]
FIB-4_{clean}	Control	0.567 [-1.334,2.468]	0.068 [-0.042,0.177]
	ATTR-CA	0.059 [-0.059,0.177]	0.081 [-0.052,0.215]
	AL-CA	-0.007 [-0.065,0.051]	0.027 [-0.035,0.089]
tr-v_{max}	Control	-0.982*** [-1.258,-0.705]	-0.156*** [-0.251,-0.062]
	ATTR-CA	-0.080 [-0.447,0.287]	-0.206 [-0.625,0.214]
	AL-CA	0.539** [0.027,1.052]	0.413** [0.020,0.806]
LASSO-selected controls		AP, TAPSE, AL-CA, ATTR-CA, active disease	AP, TAPSE, AL-CA, ATTR-CA, active disease,
constant		13.799 [-7.313,34.912]	1.772*** [0.578,2.967]

95% confidence intervals in brackets; * $p < 0.1$, ** $p < 0.05$, *** $p < 0.01$

Each of the two columns represents a multivariate regression of the logged liver stiffness on the core variables and the high dimensional controls selected by post cluster LASSO. The core variables of interest were septum thickness, NT-proBNP, PDRpeak and dilation of liver veins within both models pre-selected based on Figure 1. Coefficients for liver vein dilation(#) were not identified as there was no patient with dilated hepatic veins among the control group. The inclusion of tr-v_{max} in the models resulted in computational instability (##). The control group used in model 1 included only the subset of control patients with localized amyloidosis without cardiac impairment whereas model 2 referred to the entire control group with a high percentage of patients suffering form other cardiac disorders.
